# Intestinal Microbiome and Atherosclerosis – Authors' Reply

**DOI:** 10.1016/j.ebiom.2016.10.039

**Published:** 2016-10-27

**Authors:** Lin Chen, Tomoaki Ishigami

**Affiliations:** Yokohama City University of Graduate School of Medicine, Department of Medical Science and Cardio-Renal Medicine, 3-9, Fukuura, Kanazawa-ku, Yokohama, Japan

We highly appreciate the Commentary by Spence (Spence, 2016) on our article entitled “Commensal microbes-specific activation of B2 cell subsets contribute to atherosclerosis development independent of lipid metabolism” (Chen et al., in this issue). Atherosclerotic diseases are systemic disorders and one of the leading causes of mortality and morbidity throughout the world. Although multiple risk factors, such as hypertension, diabetes mellitus, hyperlipidemia and smoking, have been identified, atherosclerosis is considered to be initiated and sustained by various metabolic factors (Mihaylova et al., 2012, Kearney et al., 2008). Previous studies have revealed the pathway linking dietary food intake, intestinal microflora and atherosclerosis. These studies indicated that increasing the dietary intake of precursors of toxic metabolic products of the intestinal microbiome, such as the trimethylamine-*N*-oxide from phosphatidylcholine and other forms of choline and carnitine were associated with CVD (Wang et al., 2011, Koeth et al., 2013, Tang et al., 2013, Spence et al., 2016). Therefore, to conquer atherosclerotic diseases, appropriate food restrictions accompanying appropriate medical treatment are essential life-style modification requirement accepted commonly in clinical healthcare guidelines. Current progress in this area enabled us to recommend the subjects with high risk in atherosclerosis more targeted food restriction under specific diseased conditions including CKD^6^. Additionally, atherosclerosis is considered to arise from an inflammatory process. Inflammation associated with atherosclerosis involves complicated processes, including systemic inflammatory reactions and the accumulation of immune cells, such as monocytes/macrophages, dendritic cells, and lymphocytes (Shimada, 2009). The immune system, encompassing both innate immunity and adaptive immunity (cellular immunity and humoral immunity), has been implicated in all stages of atherosclerosis from initiation through progression, as well as in atherothrombotic complications (Hansson, 2009). Our current analyses with previous reports (Ishigami et al., 2013) shed a light on persistent inflammatory process in atherosclerosis focusing on pathological humoral immunity between commensal microbes and activated sub-populations of substantial B cell in the vicinity of arterial adventitia. Because atherosclerosis is becoming global health burden throughout the world especially in developed counties, multidisciplinary therapeutic and preventive approaches should be expected. In addition in future studies using our model, it would be of interest to evaluate the effects of both increasing the dietary intake of precursors of toxic metabolic products of the intestinal microbiome and abnormally enhanced humoral immunity on the development of atherosclerosis. Such effects might act independently of or perhaps be mediated by the B2-cell effects on atherosclerosis that we have described.

## Funding Sources

T.I. is supported by a Grant-in-Aid for Scientific Research from the Ministry of Education, Culture, Sports, Science, and Technology (MEXT) no. 26461257, and Yokohama Foundation for Advancement of Medical Science. L.C. is supported by MEXT Government Scholarship no. 122229. These funding sources had no role in the study design; in the collection, analysis and interpretation of data; in the writing of the manuscript; or in the decision to submit the paper for publication.

## Conflicts of Interest

The authors declare no conflict of interest.
